# Cardiac Postpneumonectomy Syndrome

**DOI:** 10.1016/j.atssr.2025.06.003

**Published:** 2025-06-25

**Authors:** Kathryne Holmes, Mouchammed Agko, John Kuckelman, Daniel Miller

**Affiliations:** 1 Department of Surgery, Medical College of Georgia, Augusta, Georgia; 2 Division of Plastic Surgery, Department of Surgery, City of Hope, Duarte, California; 3 Section of Thoracic Surgery, Department of Surgery, Medical College of Georgia, Augusta, Georgia

## Abstract

Postpneumonectomy syndrome (PPS) is a rare postoperative condition. We report a patient with cardiac PPS caused by unique cardiac-related anatomic changes. A 56-year-old man with atypical carcinoid of the right lung underwent a right intrapericardial pneumonectomy. At the 12-month follow-up, the patient complained of progressive dyspnea. Imaging demonstrated the right diaphragm elevation with significant mass effect on the right side of the heart without tracheobronchial abnormalities. A redo right thoracotomy was performed with reduction of intrathoracic contents, diaphragmatic plication, and placement of an intrathoracic tissue expander with complete correction of the anatomical abnormality and resolution of symptoms.

Postpneumonectomy syndrome (PPS) is an extremely rare condition that occurs in a small percentage of patients who undergo a pneumonectomy for a variety of reasons. The condition is characterized by intermittent dynamic airway obstruction secondary to excessive mediastinal shift. This condition affects mostly young adults and presents with progressive shortness of breath, dyspnea, and stridor as well as recurrent respiratory infections.^1^ The objective of this case report is to highlight the unique anatomic changes seen in our patient 12 months after an intrapericardial pneumonectomy. The intrathoracic changes that caused our patient’s symptoms were secondary to ipsilateral diaphragm atrophy with intrathoracic herniation of the liver compressing the right atrium and ventricle causing cardiopulmonary compromise without tracheobronchial abnormalities.

The patient is a 56-year-old man, former smoker, who had stage IIIA atypical carcinoid of the right lung. He underwent a right intrapericardial pneumonectomy and resection of a portion of the pericardium and phrenic nerve due to tumor invasion and adjuvant chemotherapy. Six months after surgery, he started to experience shortness of breath, worsening dyspnea on exertion, and tachycardia with exercise.

A surveillance chest computed tomographic (CT) scan at the 12-month follow-up showed no evidence of recurrent cancer; however, it did show elevation of the right diaphragm with nearly complete occupation of the right chest with the liver and mass effect on the right side of the heart (Figure 1). Pulmonary function test results showed forced expiratory volume in 1 second of 52% and diffusion capacity of the lung for carbon monoxide of 72%. A 2-dimensional echocardiogram showed significant compression of the right atrium and right ventricle, with an ejection fraction of 0.50.

**Figure 1:**
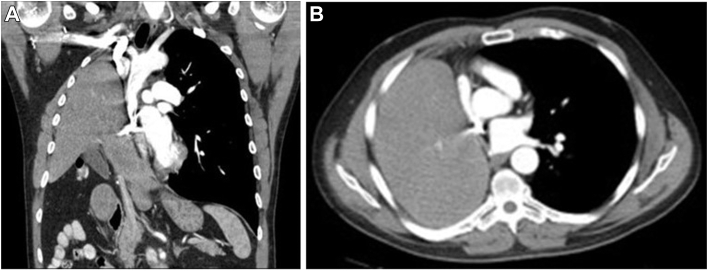
Preoperative chest computed tomographic scans. (A) Coronal image shows elevated right diaphragm with complete occupation of the pleural space with the liver and intrabdominal contents. (B) Axial image shows the intrathoracic liver exerting significant compression on the right atrium.

The patient’s symptoms were related to compression of the right atrium and ventricle without classic PPS tracheobronchial abnormalities. The patient underwent a redo right thoracotomy with reduction of intrathoracic contents, diaphragm plication, and placement of intrathoracic saline tissue expander (750 cm^3^). The hospital stay was 3 days.

One month the cardiac PPS was corrected, the patient had complete resolution of his symptoms. A postoperative CT scan showed the tissue expander in an advantageous position, without significant diaphragmatic elevation and no cardiac compression (Figure 2).

**Figure 2:**
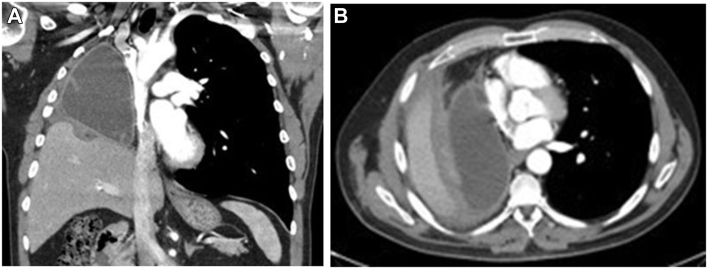
Postoperative chest computed tomographic scans. (A) Coronal image shows the advantageous position of the tissue expander with reduction of the diaphragm. (B) Axial image shows the tissue expander and improved cardiac silhouette, without compression.

## Comment

PPS is a rare and sometimes deadly complication after pneumonectomy.^2^ It can develop months to years after surgery and affects mostly children or young adults.^3^ The syndrome is characterized by mediastinal shift toward the pneumonectomy space with rotation of the great vessels and surrounding structures. This shift causes 2 distinct processes: the first is a significant mediastinal shift with hyperexpansion of the remaining lung, and the second, compression of the distal trachea and/or mainstem bronchus over the spine, results in intermittent airway compression and dynamic obstruction.^4^ This resultant airway obstruction can cause dyspnea on exertion, worsening shortness of breath, stridor, and even recurrent pulmonary infections.^3^

Once a diagnosis of PPS is suspected, further investigation must be performed. A CT scan of the chest is the imaging method of choice to confirm the diagnosis and allow recognition of the point of airway obstruction.^2^ Preoperative pulmonary function tests most commonly demonstrate airway obstruction with reduction of peak flow. Finally, bronchoscopy is instrumental in not only diagnosis but also allows for evaluation of the anatomy and the degree of obstruction and helps identify which portion of the tracheobronchial tree is malacic.^2^

Numerous surgical techniques for management have been discussed. The most popular and successful method is correction of the mediastinal repositioning with a tissue expander placement. One systematic review found 75% of patients who underwent correction with prosthesis had excellent results. The most common long-term complication is implant leakage.^5^ The success of this treatment is attributed to the ability of the implant to adapt its shape inside the chest cavity, the low pressure of the implant, and the long-term durability of the material.

Other methods include placement of expandable metallic stents and mediastinal graft fixation.^2^^,^^4^^,^^5^ These methods have not had as much success as tissue expander placement and have a higher rate of complications. In the same systematic review, complications developed in 50% of patients who underwent metallic stent placement, including stent migration, obstruction, and respiratory infections.^2^^,^^4^^,^^5^

Our case of cardiac PPS is unique in that it depicts cardiac compression caused by an elevated diaphragm and intrathoracic viscera without any airway abnormality. The diagnosis was made with CT scan, pulmonary function tests, and bronchoscopy. An echocardiogram also aided in the diagnosis of cardiac PPS, showing compression of the right atrium and ventricle. The treatment was surgical, consisting of diaphragm plication and placement of a saline prosthesis to establish nearly normal volume of the thoracic cavity and relief of the cardiac compression.

PPS is a rare complication occurring after pneumonectomy. Our report of cardiac PPS demonstrates another possible anatomical abnormality that can develop in these patients. More research is needed so we can diagnose these patients earlier or develop methods during the index procedure to minimize the risk of PPS occurrence and now cardiac PPS.
